# The relationship between serum triglyceride levels and acute pancreatitis in an animal model and a 14-year retrospective clinical study

**DOI:** 10.1186/s12944-019-1126-0

**Published:** 2019-10-23

**Authors:** Qiyue Zhang, Mengbin Qin, Zhihai Liang, Huali Huang, Yongfeng Tang, Lingyan Qin, Zhenping Wei, Mengtao Xu, Guodu Tang

**Affiliations:** 1 Department of Gastroenterology, First Affiliated Hospital, Guangxi Medical University, Nanning, 530021 Guangxi China; 2 Department of Gastroenterology, Second Affiliated Hospital, Guangxi Medical University, Nanning, 530007 Guangxi China

**Keywords:** Hypertriglyceridemia, Acute pancreatitis, Severity, Animal model

## Abstract

**Objectives:**

The aim of the current study was to evaluate influence of serum triglyceride levels on the course of acute pancreatitis (AP).

**Methods:**

Rats models of hypertriglyceridemic were used in animal experiments. Following induction of acute pancreatitis, amylase, and pancreas histological scores were all compared. In addition, in a clinical study, clinical data were collected from 1681 AP patients admitted from 2003 to 2016 who were divided into 4 groups based on their serum triglyceride (TG) levels. The clinical features among these 4 groups were compared, and a receiver operating characteristic (ROC) curve analysis was also performed on TG values to estimate their relationship with severity.

**Results:**

In animal experiments, the hypertriglyceridemic pancreatitis (HTGP) group had markedly higher serum amylase, and histological scores relative to the other animal groups. In the clinical study, we identified significant differences in gender, age, body mass index (BMI), cost, and incidence of partial complications among the 4 TG-based groups. Importantly, the TG levels on day 3–4 after admission could be used to accurately predict disease severity.

**Conclusions:**

Hypertriglyceridemia (HTG) can aggravate pancreatic injury, and hypertriglyceridemia patients are more likely to suffer from severe pancreatic injury with a higher possibility of complications. In addition, triglyceride levels are correlated with the severity of AP positively.

## Introduction

Acute pancreatitis (AP), an inflammatory illness of the pancreas, is the leading cause of hospital admission for gastrointestinal illnesses in many countries [1]. Hypertriglyceridemia (HTG) is the second most common cause of acute pancreatitis, after gallstones, accounting for 10.36% of AP cases [2]. The etiology of gestational pancreatitis in particular is thought to be attributable to HTG in approximately 56% of patients [3]. The percentage of cases in which hypertriglyceridemic pancreatitis (HTGP) results in AP is rising globally [4, 5].

Although many studies have concentrated on the relationship between AP etiology and HTG, the exact relationship between disease severity and HTG has not been fully confirmed, and the role of HTG in modulating AP disease course remains controversial [6]. Disease complications, such as renal failure, shock, infection, and mortality have been found in a number of studies to be higher in the HTG group than in controls, indicating that HTG exacerbates severe acute pancreatitis (SAP), resulting in systemic complications and an elevated SAP mortality rate [7, 8]. Kimura et al. also found that triglyceride**(**TG) levels were associated with a worsening of pancreatic injury in animal models, with extensive lipase activity and histological damage occurring in an experimental hyperlipidaemic pancreatitis model system [9, 10]. This led us to hypothesize that a similar pathophysiology may occur in the context of HTG-related pancreatitis. In addition, Tariq et al. found that a TG level ≥ 2.26 mmol/L on admission in AP patients was an independent predictor of the development of systemic and local complications, hospital length of stay, admission to the intensive care unit (ICU), and ICU length of stay [11]. Additional studies, however, found no relationship between TG levels and AP severity, rates of complications, amylase levels, or APACHE II and Ranson’s criteria score [12–14]. While these results are inconsistent with one another, they come from studies with small sample sizes potentially accounting for this discrepancy. We therefore conducted the present study with the aims of assessing the differences in the pancreas tissue damage between AP secondary to HTG and other forms of AP in rats, of assessing the clinical connection between AP disease course and variations in TG levels, and of ascertaining the role of elevated TG levels on the prognosis and severity of AP. To this end, we assessed a wide range of collected parameters in a series of 1681 patients from the First Affiliated Hospital of Guangxi Medical University over a 14-year period. We further classified AP patients into specific subgroups to investigate thoroughly the relationship of TG levels and AP severity, and we used animal models to gain mechanistic insights into the role of TG in pancreatitis-associated damage.

## Materials and methods

### Animal experiments

Four-week-old male SD rats (70–80 g) were obtained from the Experimental Medical Center of Guangxi Medical University. All rats were maintained in a temperature-controlled room (22 ± 1 °C). Rats were divided into three groups at random (*n* = 6 per group): Control rats (NORMAL), rats with hypertriglyceridemic pancreatitis (HTGP), and rats with non-hypetriglyceridemic pancreatitis (NHTGP). The HTGP rats were fed a high fat diet, while the NORMALrats and the NHTGP rats were fed a normal fat diet for 4 weeks. The high fat diet formula consisted of 15% lard, 2% cholesterol (Weijia, China), 0.2% sodium cholate (Weijia, China), and 82.8% normal fat diet (Kaoxieli, China) forage. After 4 weeks feeding, triglyceridemia levels were quantitated using blood samples collected from the posterior orbital plexus of all rats in this three groups tested by Hitachi 7600 automatic biochemical analyzer (Hitachi, Japan) in clinical laboratories of the First Affiliated Hospital of Guangxi Medical University. Detection method is GPO-PAP method by reagent kit for triglycerides test (Denuo, China). All the animals were fasted for 12 h prior to surgical operations. An AP model was established in the rats in HTGP and NHTGP groups via intraperitoneal injection of caerulein (50 μg/kg) (Sigma Aldrich, USA) every 1 h for 7 consecutive hours. Normal control rats received saline injections at the same time points. After 24 h, rats were euthanized [15]. Serum amylase levels were quantitated in EPS method (α-glucosaccharase) by reagent kit for α-amylase test (Denuo, China) using blood samples collected from the right atrium of all rats. Serum amylase levels were also tested by Hitachi 7600 automatic biochemical analyzer (Hitachi, Japan) in clinical laboratories of the First Affiliated Hospital of Guangxi Medical University. Pancreatic tissue samples were collected from all rats following euthanasia. A section of the pancreatic gland was fixed using 10% formalin, paraffin-embedded, and used for morphologic assessment by H&E staining. The degree of pancreatic injury was assessed by light microscopy based on the severity of edema, hemorrhage, inflammatory cell infiltration, and cell destruction. The pathologic scores of these samples were evaluated by a pathologist pursuant to Schmidt’s criteria on the severity of these conditions [6, 10]. TG levels, serum amylase, and pancreatic tissue histological scores were then compared among these three experimental groups.

### Clinical study

#### Patients

In the clinical study, we collected clinical data from 2670 AP patients admitted between 1 June 2003 and 1 June 2016 to the First Affiliated Hospital of Guangxi Medical University. The diagnosis of AP was confirmed based on revised Atlanta Criteria prior to using subject data in the present study. A diagnosis of AP necessitated two of the following three attributes: (1) abdominal soreness; (2) serum lipase activity (reference range, 0–60 U/L) or amylase activity (reference range, 0–220 U/L) at least three times grander than the upper limit of normal; (3) typical facilities inspection of acute pancreatitis such as contrast-enhanced computed tomography (CECT) or less commonly magnetic resonance imaging (MRI) or transabdominal ultrasonography [4]. Patient exclusion criteria were: (1) Patients who were < 18 years old or pregnant were excluded from this analysis; (2) patients diagnosed as possessing chronic pancreatitis were likewise eliminated.

#### Serum triglyceride gradation

Serum triglyceride levels were detected in a total of 1681 patients within 48 h of hospital admission. These 1681 AP patients were retrospectively parceled out into 4 groups, including a normal triglyceride group (serum TG levels of 0–1.7 mmol/L), [16] a mild hypertriglyceridemia group (serum TG levels of 1.7–5.6 mmol/L), a moderate hypertriglyceridemia group (serum TG levels of 5.6–11.2 mmol/L), and a severe hypertriglyceridemia group (serum TG levels of ≥11.2 mmol/L).

#### Data collection and clinical manifestations

The biochemical parameters collected from patient datasets were obtained either upon the initial presentation or within 10 days of hospital admission. Organ dysfunction was defined as reported by the Atlanta classification; severe pancreatitis was diagnosed when there were indications of dysfunction in other organs [4]. Data were collected on the patients’ critical gestures at admission, including hemogram, blood biochemical parameters, and imaging results including those from CECT, MRI, or transabdominal ultrasonography. Baseline clinical characteristics from patients including age, gender, weight, height, body mass index (BMI), diabetes, levels of white blood cells (WBC), Serum lipase (LPS), amylase (AMY), calcium concentration (Ca^2+^), C-reactive protein (CRP), and glucose (GLU) determined within 48 h of admission were used for statistical analyses in this study. In addition, instances of pancreatic effusion, pancreatic pseudocyst, pancreatic necrosis, pancreatic abscess, abdominal compartment syndrome (ACS), acute respiratory distress syndrome (ARDS), kidney injury, hydropericardium, arrhythmia, pancreatic encephalopathy (PE), disseminated intravascular coagulation (DIC), septicaemia, fungal infection, chronic pancreatitis, hyperglycemia, and hydrothorax, were recorded and compared among the 4 TG groups. Furthermore, a receiver operating characteristic (ROC) curve analysis was also implemented on the TG values to determine SAP.

### Statistical analyses

Statistical analyses were conducted using SPSS v20.0 (Statistical Package for Social Sciences, Chicago, USA). Numerical data are expressed as the means ± SEM, and categorical variables are expressed as the means (ratios). For comparisons of continuous variables t-tests and ANOVAs were used as appropriate. Categorical variables were evaluated using a χ^2^ test, and Fisher’s exact test was used when the number of observations was < 5. Serum TG was evaluated by means of a receiver operating characteristic curve for selected cut-off points. *P* <  0.05 was considered with statistically significance.

## Results

### Animal experiments

In our animal experiments, we observed markedly higher TG levels in rats in the HTGP group, which also had elevated serum amylase and pancreas histological scores relative to rats in the NHTGP group. The mean serum TG levels in the HTGP group were significantly elevated relative to the NHTGP group (*P* <  0.05) (Table 1). Similarly, pancreas histology scores were higher in HTGP rats relative to NHTGP rats, whereas serum amylase levels were significantly higher in both AP groups relative to normal controls (all *P* <  0.05; Table 1).

**Table 1:** Triglyceride, Serum Amylase and Pancreatitis Histological Score in Rats of 3 Groups

	NORMAL (*n* = 6)	NHTGP (*n* = 6)	HTGP (*n* = 6)	*P*
TG (mmol/L)	0.99 ± 0.30	0.99 ± 0.30	3.11 ± 0.73	< 0.05
AMY(U/L)	1443.29 ± 61.39	1886.83 ± 84.78	2119.00 ± 86.24	< 0.05
Histological scores of pancreas	0.00	4.50 ± 1.52	6.43 ± 1.27	< 0.05

**Fig. 1:**
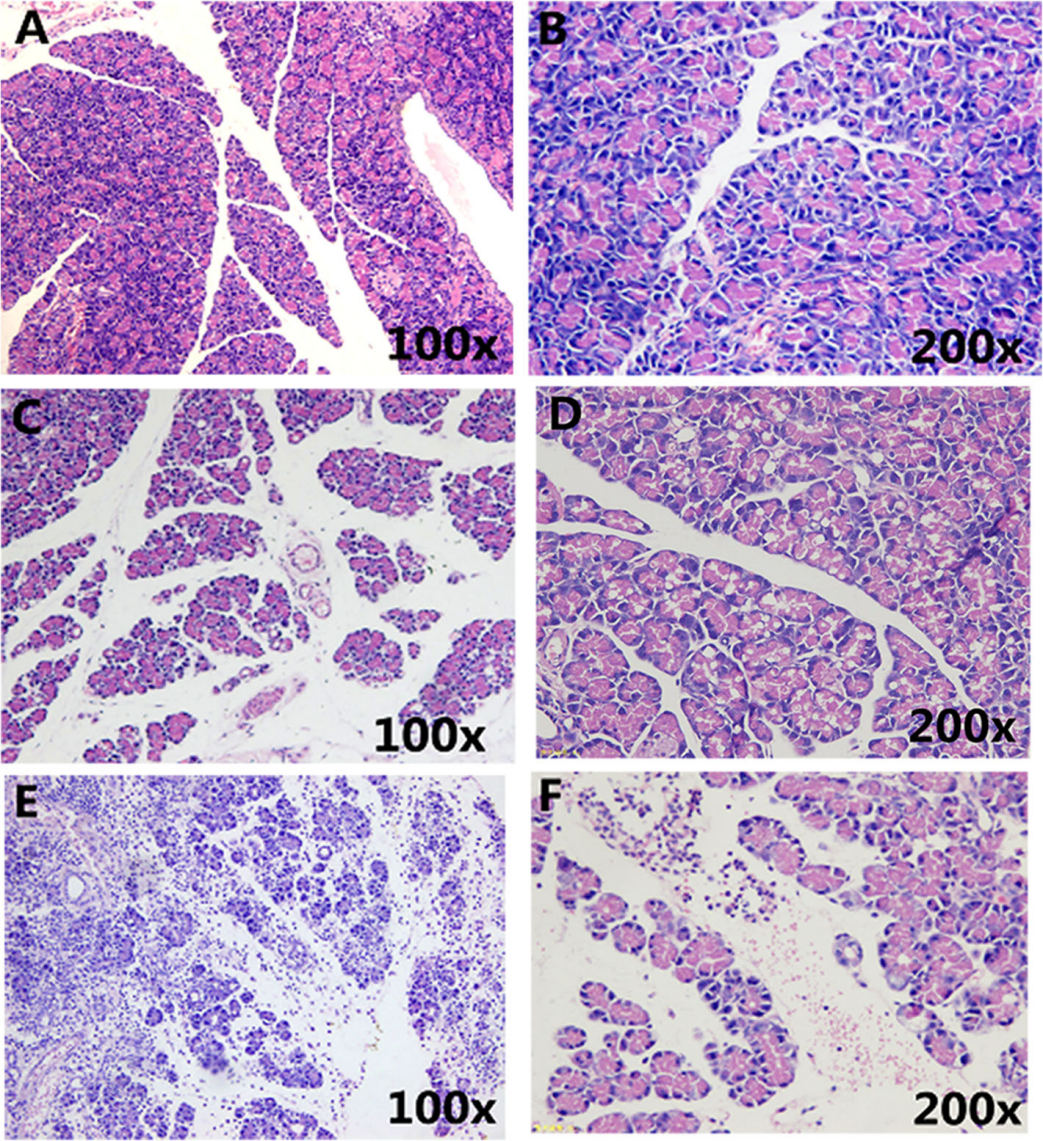
Pancreatic Pathology. **a**, **b** Normal group pancreas sections; (**c**, **d**) Pancreas sections for rats in the NHTGP group. The space between pancreatic lobules was significantly wider than in normal control rats, with clear edema and a large number of alveolar cells were vacuolar; (**e**, **f**) In the HTGP group, a large number of inflammatory cells infiltrated and exudate were evident in the pancreatic parenchyma, with evidence of pancreatic parenchymal hemorrhage

Pancreatic necrosis was not observed in any group, with negligible changes in pancreatic histology in the normal group. Both the HTGP and NHTGP groups had substantial evidence of pancreatic edema and inflammatory cells infiltration, acinar cells vacuoles, and hemorrhage. Rats in the HTGP group exhibited more substantial pancreatic impairment than did those in the NHTGP group (Fig. 1).

### Clinical study

#### Basic patient demographics

Baseline patient demographics for patients divided based on TG levels are shown in Table 2.

**Table 2:** Baseline Characters of Patients Versus Serum Triglyceride

	Normal TG	Mild HTG	Moderate HTG	Severe HTG	*P*
Sum	1018 (60.6%)	434 (25.8%)	127 (7.6%)	102 (6.1%)	–
Gender	625 (61.5%)	346 (79.9%)	105 (82.7%)	83 (81.4%)	0.000
Diabetes	61 (6.4%)	45 (11.2%)	26 (22.8%)	24 (26.4%)	0.000
Age (year)	51.77 ± 0.55	45.88 ± 0.73	40.03 ± 0.88	43.53 ± 1.04	0.000
Weight (kilogram)	61.01 ± 1.71	69.50 ± 3.54	108.68 ± 18.61	89.38 ± 17.02	0.000
Height (centimeter)	162.70 ± 0.90	163.08 ± 0.71	166.02 ± 1.03	166.57 ± 0.90	0.370
BMI (kg/m^2^)	22.43 ± 0.18	23.86 ± 0.27	26.77 ± 0.62	25.71 ± 0.55	0.000

This clinical study included 1681 AP patients; of these, 1018 (60.6%) had normal TG levels (0.92 ± 0.01 mmol/L), 434 (25.8%) had mild HTG levels (3.00 ± 0.05 mmol/L), 127 (7.6%) had moderate HTG levels (7.88 ± 0.13 mmol/L), and 102 (6.1%) had severe HTG levels (18.59 ± 0.57 mmol/L). Patients with mild, moderate, or severe HTG were more commonly male (61.5% vs. 79.9% vs. 82.7% vs. 81.4%, respectively; *P* < 0.05), younger (mean age in years 51.77 ± 0.55 vs. 45.88 ± 0.73 vs. 40.03 ± 0.88 vs 43.53 ± 1.04; *P* < 0.05), and fatter (mean BMI 22.43 vs. 23.86 vs. 26.77 vs. 25.71; *P* < 0.05) compared to patients with typical TG levels. Histories of diabetes mellitus (6.4% vs. 11.2% vs. 22.8% vs. 26.4%; *P* < 0.05) were also more common in HTG groups.

#### Comparison of laboratory parameters upon admission among patients with different TG levels

Some parameters, such as AMS, LPS, Ca^2+^, CRP, and GLU exhibited significant differences between groups (*P* < 0.05). Significant differences were not discovered in WBC, NEU values between the 4 groups (*P* > 0.05; Table 3).

**Table 3:** Initial Laboratory Values of Patients in 48 Hours Versus Serum Triglyceride

	Normal TG	Mild HTG	Moderate HTG	Severe HTG	*P*
TG (mmol/L)	0.92 ± 0.01	3.00 ± 0.05	7.88 ± 0.13	18.59 ± 0.57	0.000
WBC(10^9^/L)	11.87 ± 0.20	12.14 ± 0.25	12.06 ± O.39	12.43 ± 0.37	0.744
NEU(%)	59.20 ± 1.13	61.33 ± 1.59	56.67 ± 3.26	53.82 ± 3.80	0.197
AMS(U/L)	662.85 ± 24.68	339.05 ± 25.85	414.86 ± 58.78	500.18 ± 54.64	0.000
LPS(U/L)	361.19 ± 17.11	190.27 ± 16.73	200.97 ± 40.53	358.28 ± 62.93	0.000
Ca^2+^(mmol/L)	2.13 ± 0.00	2.11 ± 0.01	2.01 ± 0.02	1.94 ± 0.04	0.000
CRP (mg/L)	74.33 ± 4.07	102.72 ± 8.37	169.60 ± 16.02	140.64 ± 16.75	0.000
GLU (mmol/L)	8.11 ± 0.44	8.65 ± 0.37	28.26 ± 11.00	12.77 ± 0.64	0.000

Reference ranges: TG, 0–1.7 mmol/L; WBC, 3.5–9.5 × 10^9^/L; NEU, 40–75%; AMS, 0-220 U/L; LPS, 0-60 U/L; Ca^2+^, 2.0–2.7 mmol/L; CRP,0.00–5.00 mg/L; GLU, 3.9–6.1 mmol/L

#### Clinical outcomes

Table 4 shows the complications developed by all study patients with AP. In the four TG subgroups (normal to severe), pancreatic effusion was observed in 419 (43.3%), 237 (58.7%), 98 (79.7%), and 77 (78.6%) patients (*P* < 0.05). Pancreatic necrosis was the next most common complication (13.3% vs. 28.6% vs. 20.6% vs. 32.7%), followed by ACS (0.4% vs. 1.5% vs. 4.8% vs. 2.0%), ARDS(2.7% vs. 5.5% vs. 11.0% vs. 9.8%), acute renal failure (3.8% vs. 6.5% vs. 10.2% vs. 18.6%), DIC(0.5% vs. 0.2% vs. 1.6% vs. 2.9%), and hydrothorax (32.4% vs. 43.5% vs. 54.7% vs. 60.6%). No significant difference in the incidence of pancreatic pseudocyst, hydropericardium, arrhythmia, pancreatic encephalopathy, septicaemia, fungal infection, chronic pancreatitis, or hypergemia were observed between these 4 groups (*P* ≥ 0.05).

**Table 4:** Clinical Manifestation of Patients Versus Serum Triglyceride

	Normal	Mild HTG	Moderate HTG	Severe HTG	χ^2^	*P*
Pancreatic effusion	419(43.3%)	237 (58.7%)	98 (79.7%)	77 (78.6%)	101.78	0.000
Pancreatic pseudocyst	92 (9.5%)	44 (10.6%)	11 (9.0%)	4 (4.1%)	3.92	0.264
Pancreatic necrosis	129(13.3%)	84 (28.6%)	49 (20.6%)	32 (32.7%)	69.74	0.000
Pancreatic abscess	15 (1.5%)	10 (2.4)	6 (4.8%)	4 (4.1%)	7.74	0.035
ACS	4 (0.4%)	6 (1.5%)	6 (4.8%)	2 (2.0%)	16.81	0.001
ARDS	27 (2.7%)	24 (5.5%)	14 (11.0%)	10 (9.8%)	28.50	0.000
Kidney injury	39 (3.8%)	28 (6.5%)	13 (10.2%)	19 (18.6%)	42.04	0.000
Hydropericardium	12 (1.2%)	6 (1.4%)	3 (2.4%)	0 (0%)	2.35	0.468
Arrhythmia	7 (0.7%)	3 (0.7%)	2 (1.6%)	0 (0%)	2.06	0.577
PE	3 (0.3%)	3 (0.7%)	1 (0.8%)	2 (2.0%)	5.58	0.089
DIC	5 (0.5%)	1 (0.2%)	2 (1.6%)	3 (2.9%)	8.97	0.018
Septicaemia	2 (0.2%)	2 (0.5%)	1 (0.8%)	1 (1.0%)	4.14	0.181
Fungal infection	5 (0.5%)	4 (0.9%)	2 (1.6%)	0 (0%)	3.03	0.296
Chronic pancreatitis	18 (1.8%)	8 (1.9%)	2 (1.7%)	0 (0%)	1.348	0.740
Hyperglycemia	55 (5.6%)	36 (8.7%)	11 (9.6%)	13 (14.0%)	12.776	0.005
Hydrothorax	301(32.4%)	170 (43.5%)	64 (54.7%)	57 (60.6%)	50.816	0.000

No significant differences were noted in the development of mortality among these groups, as shown in Table 5. AP patients exhibited a higher frequency of severe AP with increasing TG levels (28.3% vs. 37.4% vs. 54.8% vs. 51.0%) (*P* < 0.05). Hospitalization expenses also increased as TG levels increased (30,186.21 ± 1664.03 vs. 33,345.25 ± 3035.53 vs. 38,210.34 ± 5658.28 vs. 48,336.83 ± 9338.23) (*P* < 0.05).

**Table 5:** Severity and Outcomes of Patients Comparison Versus Serum Triglyceride

	Normal HTG	Mild HTG	Moderate HTG	Severe HTG	χ^2^	*P*
SAP	281(28.3%)	161(37.4%)	68 (54.8%)	50(51.0%)	53.09	0.000
Death	67(6.6%)	40 (9.2%)	10 (7.9%)	11 (10.8%)	4.50	0.212
Expense	30,186.21 ± 1664.03	33,345.25 ± 3035.53	38,210.34 ± 5658.28	48,336.83 ± 9338.23	–	0.020

#### ROC analysis of TG levels to predict severe acute pancreatitis

TG levels on days 1–2, 3–4, 5–6, 7–8, and 9–10 were compared between the non-severe acute pancreatitis group (NSAP) and severe acute pancreatitis group (SAP). TG levels in SAP patients were much higher than in NSAP patients on days 1–6 post admission (*P* < 0.05; Table 6).

**Table 6:** Different Values of TG in NSAP and SAP Cases on DAY1–2,3-4,5-6,7–8, and 9–10 (Mean ± SEM)

Characterictic		NSAP	SAP	*P*
TG, mmol/L	DAY 1–2	2.53 ± 0.12	4.04 ± 0.23	0.000
	DAY 3–4	1.85 ± 0.27	4.35 ± 0.55	0.000
	DAY 5–6	2.65 ± 0.24	3.55 ± 0.32	0.041
	DAY 7–8	3.65 ± 1.13	3.68 ± 0.45	0.975
	DAY 9–10	2.31 ± 0.40	2.85 ± 0.34	0.515

**Fig. 2:**
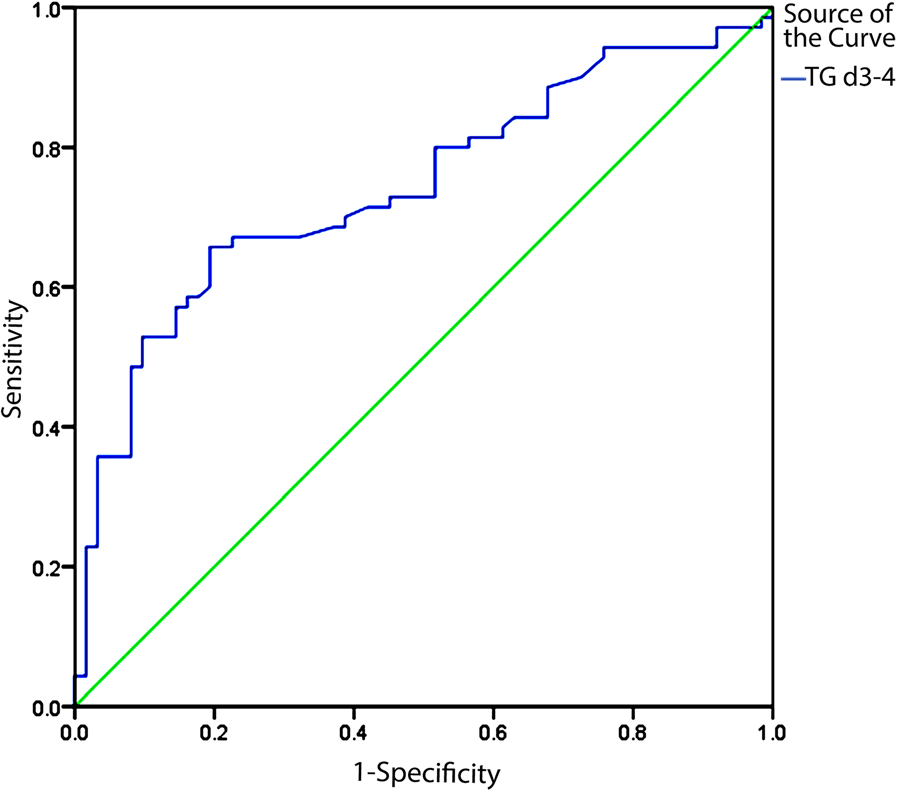
ROC Analysis of Day 3–4 TG Levels as a Means of Predicting Severe Acute Pancreatitis

We therefore performed a ROC analysis of patient TG levels on days 1–6 in an effort to identify values predictive of SAP. The area under the ROC curve in the prediction of SAP on days 0–2, days 3–4, and days 5–6 was 0.609 vs. 0.742 vs. 0.614, with significance at all tested time points (*P* < 0.05). TG levels in Day3–4 still have highest accurancy in predicting occurance of SAP which is consistent with the conclusion.TG levels on day 3–4 (with a threshold value of 2.06 mmol/L) could be used to accurately predict SAP better than could values on any other day of hospitalization. (Table 7) The ROC curve showed that a TG level of 2.06 mmol/l on day 3–4 was the optimal cut-off value for predicting more severe AP disease, with a sensitivity and specificity of 65.7 and 80.6%, respectively (Fig. 2).

**Table 7:** Most Appropriate Cut-off Points of TG in the Prediction of SAP on DAY1–2,3–4, and 5–6

Characteristics	Cut-off Points	*P*	AUC	Sensitivity	Specificity	Youden Index	PPV	NPV	Accurancy
Day1–2 TG	2.39	0.000	0.609	0.418	0.761	0.179	0.466	0.723	0.646
Day3–4 TG	2.06	0.000	0.742	0.657	0.806	0.463	0.793	0.675	0.727
Day5–6 TG	3.54	0.064	0.614	0.431	0.821	0.252	0.758	0.524	0.600

##### Reference range: TG:0.3–1.7 mmol/l, AUC indicates area under ROC curve

PPV: positive predictive value, NPV: negative predictive value.

## Discussion

Our research showed that pancreatitis that is initiated through unrelated pathogenic pathways can still be exacerbated by elevated TG grades in animal models, and the SAP rate was increased with increasing HTG severity in a clinical setting, which indicate that HTG can aggravate pancreatic injury, and that HTG patients are more likely to suffer from more severe pancreatic injury and have a higher possibility of local and systemic complications following the initial disease presentation. TG levels are also positively correlated with the severity of AP. To our knowledge, this study is the first to use a combination of animal and clinical trials in an effort to establish the relationship between serum TG levels and AP severity.

In our animal study, we established an animal model of HTG and induced acute pancreatitis via intraperitoneal injection of cerulein in rodents to further explore the relationship between HTG and AP. We observed significantly increased serum amylase levels in rats in the HTGP group, and increased pancreatic tissue inflammation relative to the NHTGP group in our animal study, consistent with previous studies, [17] Higher mean serum TG levels in our HTGP group of rats is relative to the NHTGP group, which means we establish HTG animal models in rats. And this group also showed evidence of more substantial pancreatic histological damage. In clinical data, we found that HTGP patients had higher levels of biomarkers of severe pancreatitis than did their counterparts with normal TG levels, and that HTG patients are more likely to suffer from severe pancreatic injury and a higher possibility of local and systemic complications. Levels of serum TG were closely correlated with AP disease severity, although we did not observe any significant differences in ultimate prognosis. Importantly, the TG levels of AP patients on day 3–4 post-hospitalization could be used to accurately predict SAP (with a cut-off value of 2.06 mmol/L), suggesting that HTG can aggravate pancreatic injury.

In our clinical study, patients with mild, moderate, and severe TG levels were more likely to be younger and heavier than patients with normal TG levels. The incidence of diabetes has also been found to be much greater in patients with elevated TG levels relative to patients with normal TG levels [18]. TG levels are also a key diagnostic component of identifying patients with metabolic syndrome according to the International Diabetes Federation criteria [19]. Patients with hypertriglyceridemia have a host of associations such as obesity, differing diet including alcohol, and metabolic syndrome. After the trigger is pulled, metabolic syndrome may be associated with a pro-inflammatory state which may aggravate inflammation [20]. and the presence of metabolic syndrome at admission is connected with a higher risk of severe acute pancreatitis, as well as with an elevated mortality rate and longer ICU occupancy [11, 21, 22].

When assessing laboratory parameters of AP patients within 48 h of admission, we saw that calcium levels were significantly lower in patients with higher TG levels, and these patients also had elevated blood pressure and CRP consistent with previous work. Indeed, CRP is a common marker of disease severity with good prognostic accuracy for severe acute pancreatitis [23, 24]. Higher blood glucose is indicative of endocrine pancreatic insufficiency and develops more easily during or after AP [25]. Patients in the high-TG group had lower calcium levels, which may imply that more lipotoxicity occurs in patients with HTGP with higher TG levels, leading to a poor prognosis [26]. We also found mean serum amylase to be not more than 3 times the upper limit of normal serum amylase in mild, moderate, and severe HTG groups in this study – indeed, only the normal group had a mean amylase level elevated more than 3-fold. Past research has found that normal amylase levels can be present in patients with TG levels above 5.7 mmol/L due to interference with the calorimetric reading, [27] These findings highlight the importance of physicians being aware of potentially misleadingly low levels of amylase in patients with elevated TG levels.

Hypertriglyceridaemia has been found to be linked with severe AP by our study and others [28–30]. We observed more severe pancreatitis in the high TG group in this study, implying that higher TG levels are associated with poorer prognosis in patients with HTGP. The incidence of mortality and complications was assessed in patients with AP. In these four TG subgroups, pancreatic effusion was the most differentially present complication, followed by pancreatic necrosis, abdominal compartment syndrome, acute respiratory distress syndrome, acute renal failure, disseminated intravascular coagulation, and hydrothorax. Nor were there any significant differences in mortality. AP severity and hospitalization costs were both found to increase as patient TG levels increased. Several recent studies have found that HTG is a prognostic indicator of AP-related complications such as pancreatic necrosis, pertinacious SIRS, and the need for admission to the ICU [8, 11]. Previous work thus leaves it unclear as to whether elevated TG levels are a risk factor for AP morbidity, a driver of disease etiology, or some satellite phenomenon associated with AP progression. The relationship between lipotoxicity and TG levels still warrants further investigation.

Given the clear differences in clinical outcomes among patients with varying TG levels, it is important for clinicians to identify these high risk patients. Patients with HTG presenting with AP should undergo serum TG level measurements and should undergo more frequent clinical observation and active symptomatic treatment. Those patients who will go on to develop severe pancreatitis may therefore be able to be admitted to the intensive care unit more quickly, allowing for quicker access to needed care [31]. In the long term, metabolic syndrome can be addressed in these patients through lifestyle changes and pharmacological interventions [19]. For the treatment of HTGP, it is critical to rapidly reduce TG levels and block progression of SIRS [22]. New hypolipidemic therapy is needed urgently. In addition to plasmapheresis, The potential use of alternative medicine such as anabasis aretioides, lasianthera africana, rutin and curcumin were proved lowering TG levels rapidly, making them worthy and promising strategies for clinical implementation, especially for patients with moderate or severe HTG [32–34]. In summary, to improve patient outcomes, experienced clinicians should identify severe pancreatitis based on symptoms, clinical and laboratory markers and various scoring systems as soon as possible in patients by taking risk factors into account [35]. In conclusion, serum TG levels are closely correlated with AP disease severity. HTG can aggravate pancreatic injury, and HTG patients are more likely to suffer from more severe pancreatic injury and a higher possibility of local and systemic complications. While TG levels are positively correlated with the severity of AP, no significant differences were observed in terms of prognosis among groups with different TG levels. Importantly, patient TG levels on day 3–4 post-admission (with a cut-off threshold of 2.06 mmol/L) can be used to accurately predict SAP. Future efforts at targeted therapeutic intervention will be needed to effectively reduce the mortality associated with acute pancreatitis, and early clinical recognition is vital to provide such targeted treatment and reduce rates of complications [36].

Further exploration of both a superior predictive SAP index and hypolipidemic therapy are needed. While much work has been conducted to date aimed at promoting apprehension better understanding of the pathophysiology of AP patients and of the systemic inflammation that leads to organ failure and SIRS, there still remain many unanswered questions for subsequent investigations [37].

There are several limitations to the present study. For one, this study was retrospective, potentially introducing unintended bias. In addition, we were unable to conduct comparisons between any two groups within the study, and as such we were unable to assess long-term efficacy or disease recurrence. Further randomized controlled studies and experimental work will be needed to better identify appropriate treatments for improving pancreatitis in patients such as those enrolled in the present study.

## Conclusion

HTG can aggravate pancreatic injury, and HTG patients are more likely to suffer from more severe pancreatic injury and have a higher possibility of local and systemic complications. In addition, TG levels are positively correlated with the severity of AP. It is imperative to reduce TG levels rapidly and block progression of SIRS timely during the onset of acute pancreatitis.

## Data Availability

The datasets during and/or analyzed during the current study are available from the corresponding author on reasonable request.

## Abbreviations

ACS: Abdominal compartment syndrome
AMY: Amylase
AP: Acute pancreatitis
ARDS: Acute respiratory distress syndrome
BMI: Body mass index
Ca^2+^: Calcium concentration
CECT: Contrast-enhanced computed tomography
CRP: C-reactive protein
DIC: Disseminated intravascular coagulation
GLU: Glucose
HTG: Hypertriglyceridemia
HTGP: Hypertriglyceridemic pancreatitis
ICU: Intensive care unit
LPS: Lipase
MRI: Magnetic resonance imaging
NHTGP: Non-hypetriglyceridemic pancreatitis
NSAP: Non-severe acute pancreatitis group
PE: Pancreatic encephalopathy
ROC: Receiver operating characteristic
SAP: Severe acute pancreatitis
SIRS: Systemic inflammatory response syndrome
TG: Triglyceride
WBC: White blood cells
